# Body Composition of Healthy Cats and Cats with Chronic Kidney Disease Fed on a Dry Diet Low in Phosphorus with Maintenance Protein

**DOI:** 10.3390/toxins14120865

**Published:** 2022-12-09

**Authors:** Daniela P. Machado, Bruna Ruberti, Fabio A. Teixeira, Thiago H. A. Vendramini, Karina Pfrimer, Fernanda C. Chacar, Julio C. C. Balieiro, Cristiana F. F. Pontieri, Marcio A. Brunetto

**Affiliations:** 1 School of Veterinary Medicine and Animal Science (FMVZ), University of São Paulo (USP), 225, Duque de Caxias Norte Ave, Pirassununga, São Paulo 13635-900, Brazil; 2 Nutritional Development Center, Grandfood Industria e Comercio LTDA (PremieRpet®), Luiz Augusto de Oliveira Hwy, km 204, Dourado, São Paulo 13590-000, Brazil; 3 Department of Internal Medicine, Ribeirão Preto Medical School, University of São Paulo (USP), Ribeirão Preto, São Paulo 14049-900, Brazil

**Keywords:** body condition score, feline, azotemia, lean mass

## Abstract

The aim was to evaluate the effect of feeding a low-phosphorus and maintenance protein diet in healthy cats and cats with chronic kidney disease (CKD) with IRIS stages 1 (CKD-1) and 2 (CKD-2). Cats were initially fed a senior diet (30 days) followed by the renal diet (60 days). Body composition, body weight (BW), muscle mass score (MMS), and body condition score (BCS) were assessed before (T30) and after renal diet intake (T60). General mixed linear models were used to assess the effects of fixed groups and moments (T30 × T60), as well as their interaction, in addition to the random effects of animals within each group. Unlike healthy cats and cats with CKD-1, cats with CKD-2 had a loss of BW, lower BCS (*p* < 0.005), and lower MMS (*p* = 0.0008) after 60 days of consuming the renal diet. The fat mass and lean body mass (LBM), determined by the deuterium isotopes method, did not change in all cats between T0 and T60. In healthy cats and cats with CKD-1, the renal diet resulted in maintenance of BW, BCS and MMS; but cats with CKD-2 presented lower BCS and did not reduce phosphatemia after consumption.

## 1. Introduction

Chronic kidney disease (CKD) is the most commonly recognized form of kidney disease and is a common cause of morbidity and mortality in cats [1,2]. It is defined as any structural and/or functional abnormality of one or both kidneys for three months or longer [3]. Typically, this disease is characterized by a progressive decrease in kidney function, resulting in accumulation of the parathyroid hormone and uremic toxins such as creatinine, symmetric dimethylarginine (SDMA), and urea and disruption of homeostasis [4,5].

Clinical studies of cats with CKD have demonstrated that dietary modifications can slow the disease’s progression and the development of mineral disorders, as well as prevent or control the clinical signs of uremia, increase survival time, and improve quality of life [6,7,8]. Nutritional management is essential in the treatment of CKD, and the use of specially designed diets for cats with this condition is now a scientific-based recommendation in veterinary practice [7]. Typical renal diets have reduced protein, phosphorus, and sodium content and increased caloric density, potassium, B vitamins, antioxidants, and ω-3 polyunsaturated fatty acids (PUFAs), and there are discussions about the roles of vitamin D on CKD progression [3,7,9,10]. These nutritional recommendations are indicated for all stages of CKD, with little specific information for each stage. Protein restriction has been recommended for patients with kidney disease for decades [11,12], based mainly on the premise that the accumulation of nitrogenous compounds can generate uremic crises. However, in stages 1 and 2 of CKD, the retention of nutrient compounds such as urea and creatinine are minor [13]. Recent studies have suggested that cats with CKD may require more protein than what is provided in renal diets because body weight (BW), body condition score (BCS) and/or muscle mass (MM) may decline over time [14,15]. The reason for this is that the reduced protein intake results in insufficient essential amino acid intake as well. Therefore, they would require a higher amino acid consumption [16].

Although the nutritional profile of therapeutic diets for cats with CKD has not been established, there is no disagreement between researchers and veterinarians regarding the damage caused by high phosphate ingestion. In cats with CKD, phosphorus excretion is compromised, leading to hyperphosphatemia, which is associated with the development of mineral disturbances since the early stages of CKD, along with a progressive decline in kidney function, inflammation, and oxidative stress [3,14]. In studies performed in cats with CKD, serum phosphorus concentrations were inversely associated with survival time [8,17,18].

We hypothesized that in healthy cats and cats with CKD, controlled protein consumption could maintain BW, BCS, and MM, and a low phosphorus consumption could reduce or maintain the serum phosphorus concentration. Thus, the aim of this study was to evaluate the effects of a renal diet containing protein levels recommended for adult cats and low phosphorus on the maintenance of BW, BCS, MM, and lean body mass (LBM) over a 2-month period.

## 2. Results

### 2.1. Animals and Food Consumption

**Figure 1:**
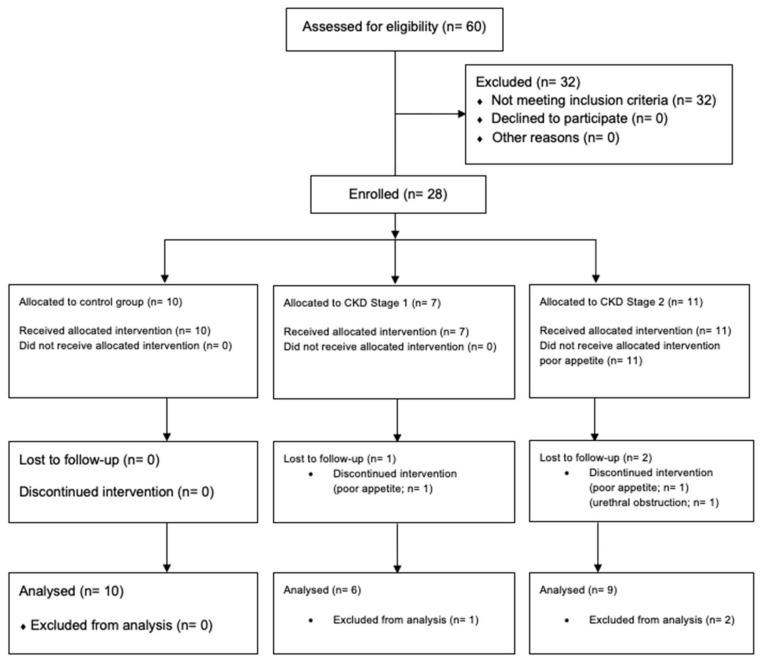
Flowchart indicating the enrollment, follow-up and analysis of the control group and CKD cats eating the renal diet.

Initially, 60 medical records of cats were analyzed, but 32 cats were excluded due to the presence of concomitant diseases and age under 1 year; therefore, 28 cats were enrolled in the study (Figure 1). Two cats with CKD stages 1 and 2 according to the International Renal Interest Society (IRIS) were dismissed due to refusal to eat the experimental diets and one due to an urethral obstruction during the study; thus, 25 cats completed the study. Of the 25 cats, 10 were healthy and were 5.30 ± 1.07 years old (range 3.0–13.0 years). Six were categorized as IRIS stage 1 (10.83 ± 1.05 years old; range 7.0–13.0 years) and nine cats as IRIS stage 2 [13] (10.22 ± 1.35 years old; range 7.0–19.0 years) (Figure 1).

The mean daily energy intake was not different between the groups, but it was higher (*p* = 0.0069) when cats were eating the senior diet (T30) when compared to the renal diet (Table 1). As expected, the ingestion of protein and phosphorus was higher (*p* < 0.0001) at baseline, since the senior diet contained 19% and 142% more protein and phosphorus, respectively, than the renal diet. There were no differences in protein and phosphorus intake between the groups (Table 1).

### 2.2. Body Composition

Both BCS and BW had an interaction of treatment and time (*p* = 0.0016 and *p* = 0.0106, respectively) and there were no differences between IRIS stage 1 cats and the control group for these variables, as shown in Table 2.

Cats with CKD stage 2 had significant loss of BW (*p* = 0.022) after 30 and 60 days consuming the renal diet, and significant lower BCS (*p* = 0.0012) and MMS (*p* = 0.0008) than the other groups. However, the mean BCS was 5 in the nine-point BCS system for cats (5/9), as described before [19]. Although there was no difference in lean mass among groups, cats with CKD stage 2 showed a loss in BW (−0.29 kg), mainly due to a loss of lean mass (−0.24 kg) between T0 and T60 (Table 2 and Table 3). The BC of the cats determined by the deuterium isotopes was not different in all groups between T0 and T60 (Table 3).

### 2.3. Blood Count and Biochemical Profile

Mean values for 15 complete blood count (CBC) analytes (red blood cells, reticulocytes, immature reticulocyte fraction, haematocrit, haemoglobin, mean corpuscular volume, mean corpuscular haemoglobin, mean corpuscular haemoglobin concentration, red cell distribution width, white blood cells, neutrophils, lymphocytes, monocytes, eosinophils, and basophils) were within the reference intervals for all cats at all time points. The mean platelet serum concentration values for healthy cats at T60 were below the reference intervals (100,000 to 518,000 µL), but these changes were not considered biologically relevant. Thus, no CBC results are shown.

Serum total protein, albumin, triglycerides, alanine aminotransferase (ALT), gamma glutamyl transferase (GGT), alkaline phosphatase (ALP), sodium, potassium, and chloride were within the reference intervals for all cats. The mean cholesterol concentration in cats with CKD stage 1 presented a time effect (higher at T0 (196.71 ± 14.48) than T30 and T60 (167.31 ± 14.48, 164.85 ± 14.49, respectively)) and was higher than the reference range, but it was not different from the other groups (Table 4). Serum total protein was higher at T0 and T30 (8.03 ± 0.186, 8.09 ± 0.186, respectively) than at T60 (7.78 ± 0.186; *p* = 0.0026).

There were no differences in concentration of serum creatinine (sCr) and blood urea nitrogen (BUN) among the time points evaluated. As expected, the concentration of sCr and BUN were higher in cats with CKD stage 2 (*p* = 0.0012 and *p* = 0.0002, respectively) when compared to the control group and cats with CKD stage 1 (Table 4).

Mean SDMA concentrations were <18 µg/dL at all time points in all cats (Table 4), and serum SDMA concentrations were different at the baseline (T0) (11.28 ± 1.41) and T60 (8.94 ± 1.39; *p* = 0.0103) (Table 4). One cat with CKD-1 (*n* = 1/6) had an increase in SDMA (from 6 µg/dL to 7 µg/dL), whereas five cats (*n* = 5/6) showed a decrease in serum SDMA concentrations. In cats with CKD stage 1, SDMA mean concentration was 11 µg/dL at baseline and 7 µg/dL at T60, as recommended by IRIS for this stage [13]. In cats with CKD stage 2, the mean concentration of SDMA was 14 µg/dL at baseline and 11 µg/dL at T60 (data not shown), as recommended by the IRIS guidelines (SDMA reference range for cats with CKD stage 2: 18 to 25 µg/dL).

The serum phosphorus concentration was not different at all time points, but it was higher in healthy cats (control group) than in cats with CKD stage 1 (*p* = 0.021). Although serum phosphorus concentrations of cats with CKD were within the reference range (2.6 to 6.0 mg/dL), the values were higher than those target values recommended by IRIS for cats with CKD stages 1 and 2 (between 2.7 mg/dL and 4.6 mg/dL) (Table 4). There was a significant time effect on Ca concentration with higher Ca at T0 (10.28 ± 0.12) than T30 and T60 (9.98 ± 0.12, 9.86 ± 0.12, respectively; *p* = 0.0024).

### 2.4. Parathyroid Hormone and Ionized Calcium

No significant differences in serum parathyroid hormone (PTH) and ionized calcium (iCa) were detected between the control and diseased cats at all time points. At baseline, only two cats with CKD stage 1 (*n* = 1) and stage 2 (*n* = 1) had PTH concentrations higher than the reference value (2.9 and 5.8 ρmol/L, respectively), but at T60 the concentrations were lower than 0.5 mmol/L for both cats, showing that, during 60 days of renal diet consumption, they did not develop secondary renal hyperparathyroidism. Despite higher values of PTH at the baseline, these cats showed normal iCa concentrations during the follow-up period (1.0 to 1.4 mmol/L). Serum PTH presented a time effect with higher levels at T0 (0.89 ± 0.13) than T60 (0.55 ± 0.123; *p* < 0.001).

## 3. Discussion

The renal diet that resulted in the intake of 8.68 g of protein and 0.12 g of phosphorus/100 kcal was able to maintain the BW, BCS, and MMS in healthy cats and cats with CKD stage 1 despite the lower energy intake (64.53 ± 6.58 kcal/BW^0.67^) recommended by the European Pet Food Industry Federation (FEDIAF) for feline maintenance [20]. However, as expected, weight and LBM maintenance was not achieved in cats with CKD stage 2, nor the lower phosphatemia in cats with CKD. Although phosphorus intake was lower at T30 and T60, the results were higher than the minimum recommendations of FEDIAF [20] nutrient recommendations for feline maintenance. The maintenance of BW and BCS is associated with adequate consumption of nutrients and daily calories. Although, in this study, the mean daily energy and crude protein intake were below the recommendations for feline maintenance by FEDIAF (75 to 100 kcal/kg^0.67^ and 6.25 g/kg BW^0.67^, respectively) [20], cats with CKD stage 1 maintained BW and BCS, which may indicate that for cats with CKD stage 1, these values may be overestimated, but it is underestimated for cats with CKD stage 2.

It is possible that this result (the maintenance of lean mass in cats with CKD stage 1 during the 60 days of the study) was a reflection on an adequate level of protein and essential amino acids intake. Concerning the consumption of essential amino acids, branched-chain amino acids (BCAA) are an important source of nitrogen for the synthesis of nonessential amino acids, such as glutamine and alanine, and may prevent protein malnutrition and muscle wasting caused by CKD [21].

A recent study that evaluated cats with CKD stages 1 and 2 that consumed three test dry foods (36.77%, 31.91%, and 25.94% protein and 0.5% phosphorus on a dry matter basis) showed that the cats maintained BW on all foods [22]. These findings were not similar to the results observed in the present study for cats with CKD stage 2.

The senior and renal food used in this study met the requirements for essential amino acids in cat food according to the Nutrient Requirements of Dogs and Cats (NRC) [23]. However, the NRC nutritional guidelines requirements are recommended for healthy animals and there are no clear guidelines on the amount of nutrients for cats with CKD. The results of this study indicate that the renal diet influenced the intake and maintenance of the BW and the MM in the control group and the cats with CKD stage 1, which is supported by the findings of BC at the time points evaluated. The decrease in MMS in cats with CKD stage 2 after the intake of the renal diet was not observed using BC by the deuterium method and may be explained by the subjectivity of the BCS and MMS assessment. The use of objective methods that can accurately determine the loss of fat and LBM in the early stages of CKD are important tools, because they allow for early nutritional intervention to ensure adequate intake of nutrients and calories, and to maintain BW and BCS close to ideal [13].

Cats with CKD commonly experience weight loss [15] as a result of a loss of appetite and reduced food intake [16]. Although the cats with CKD stage 2 maintained the amount of MM and fat mass over the 60 days of the study, they lost an average of 290 g of BW compared to the baseline. Cachexia may be associated with lower survival time, as shown in previous studies performed in cats with CKD [15,24]; therefore, the early detection of a decrease in lean body mass by precise methods could provide a better nutritional support to stabilize BW and/or delay the loss of LBM.

Hyperphosphatemia is a common complication observed in cats with CKD and may occur since the early stages [24,25]. Although the serum phosphorus concentrations of cats with CKD were within the reference range, the values were above the IRIS recommended target. It is important to note that hemolysis and lipemia may change the serum phosphorus concentration. Cats were gently manipulated at the time of blood sample collection to avoid hemolysis after at least 8 h of fasting; thus, the slight increase in serum phosphorus was probably not related to the occurrence of hemolysis or lipemia. Moreover, we followed rigorous criteria for all samples submitted to laboratorial analysis to avoid pre-analytical changes. We also excluded all cats younger than one year of age, since previous reports showed that younger cats have higher serum phosphorus concentrations than adults because of enhanced renal phosphate resorption due to the action of the growth hormone [26,27]. Despite the serum phosphorus concentrations being above the target reference range recommended by IRIS guidelines, PTH and iCa values were within the reference range, showing that cats with CKD did not develop renal secondary hyperparathyroidism after 60 days of renal diet consumption.

The limitations of this study were the absence of values of systolic blood pressure (SBP) of the cats and the short time of ingestion of the foods, as 60 days of feeding may not have been enough to decrease the levels of serum phosphorus or cause changes in body composition. As it was not possible to measure multiple determinations of SBP in the cats, and due to these limited data, the authors decided not to include this information in this study, even though we are aware that it is a risk factor for organ damage and blood pressure that is associated to the increase of proteinuria in cats with CKD, which promotes progressive renal injury [13,25]. Another point to be considered refers to ethical issues: the best study design would be the inclusion of a group of cats with CKD fed a maintenance diet to assess the progression of the disease. However, we already know that the use of a maintenance diet in this situation may negatively influence clinical outcomes and reduce the life expectancy of the cats included in this control group.

## 4. Conclusions

No changes were observed in the lean body mass and fat mass in all evaluated cats. However, cats with CKD-2 presented lower MMS. For healthy cats and cats with CKD stage 1, the test food was efficient in maintaining BW and BCS. These findings indicate that nutritional intervention and the use of a therapeutic renal diet with protein content recommended for maintenance cats and low phosphorus may be indicated for cats with CKD in the early stages, but a longer follow-up time may be required to evaluate the effect of this diet on renal function. The test food, as expected, reflected in higher energy consumption and lower consumption of protein for all groups evaluated, and despite the fact that the test food provided lower phosphorus intake, it was not efficient in decreasing the serum concentration of phosphorus in accordance with the IRIS recommended target, but it avoided the development of renal secondary hyperparathyroidism during the follow-up period.

## 5. Material and Methods

### 5.1. Location and Facilities

The study was conducted at the PremieRpet^®^ Nutritional Development Center (Dourado, Sao Paulo, Brazil) and at the Pet Nutrology Research Center (CEPEN pet) of the School of Veterinary Medicine and Animal Science of the University of São Paulo (FMVZ/USP), Pirassununga, São Paulo, Brazil. The study was performed according to the ethical principles in animal experimentation adopted by the Brazilian College of Animal Experimentation (COBEA) and the Ethical Principles in Animal Research established by the Ethic Committee on Animal Use of the School of Veterinary Medicine and Animal Science at the University of São Paulo (No. 3590220221) and the PremieRpet^®^ Nutritional Development Center (No. 088-18).

### 5.2. Diets

Two diets were used in this study: the pre-trial dry commercial diet (Senior diet; PremieR Gatos Castrados Acima dos 12 anos), a complete and balanced food designed to aid the management of senior cats; and a Renal Test diet, formulated for cats with CKD. Both were extruded at the PremieRpet^®^ Factory Unit (Dourado, São Paulo, Brazil). The chemical composition of the diets, expressed in g/100 kcal, is presented in Table 5. The metabolizable energy (ME) was estimated from the expected chemical composition, and the maintenance energy requirement (MER) was estimated as 75 kcal × kg BW^0.67^. Diets were prescribed as gram units, and the amount of food for each animal was calculated using MER and the diets’ ME. Fresh water was offered ad libitum. Cats were housed individually and were provided with regular opportunities to access the outdoor area, toys, and interaction with the staff from the research centers.

### 5.3. Animals and Feed Protocol

All cats were part of the animal colony of one of the research centers: PremieRpet^®^ Nutritional Development Center (Dourado, São Paulo, Brazil) and Pet Nutrology Research Center (CEPEN pet) of the School of Veterinary Medicine and Animal Science of the University of São Paulo (FMVZ/USP), Pirassununga, São Paulo, Brazil.

Healthy cats were included in the study based on history and normal findings of BCS, MMS, complete blood count (CBC), serum biochemical profile (total protein, albumin, creatinine, BUN, total phosphorus, total calcium, sodium, potassium, chloride, ALT, GGT, ALP, cholesterol and triglycerides) and abdominal ultrasound. The International Renal Interest Society guidelines were used for the diagnostic and staging of CKD. Clinical stable and hydrated cats with persistent creatinine levels < 1.6 mg/dL and SDMA < 18 μg/dL, showing some combination of abnormal renal ultrasound findings, isosthenuria or proteinuria (urine to protein creatine ratio >0.4), were enrolled as stage 1. Similar criteria were adopted for those cats with CKD stage 2, except by creatinine (ranging from 1.6 to 2.8 mg/dL) and SDMA levels (18 to 25 μg/dL) [18].

All the cats in the control group showed laboratory parameters within the normal range for the species. Cats with less than one year old, with comorbidities, pregnant, with BCS under 4 out of 9 [19], or with the inability to eat dry food were excluded.

### 5.4. Body Composition (BC)

The same trained veterinarian (D.P.M.) determined the BCS according to the nine-point scale [19], and MMS according to a four-point scale [26] at all moments of evaluation to reduce possible differences due to subjectivity.

The BC was determined by the method of deuterium isotopes dilution according to the methodology previously described [27]. Cats underwent 8 h of fasting and 2 h without water, then 1 mL/kg BW of deuterium oxide at 10% solution was administered subcutaneously. Blood samples of 3 mL were obtained by jugular or cephalic venipuncture before and after 2 h of deuterium oxide administration. Blood samples were processed for serum extraction and serum was stored at −20 °C until analysis.

Deuterium enrichment was determined by isotopic ratio mass spectrometry (Calixto System, Sercon Ltd, Gateway, United Kingdom) at the Isotope-ratio Mass Spectrometry Laboratory of the Internal Medicine Department of the Medical School of Ribeirão Preto –University of São Paulo. After total body water quantification, total lean mass was calculated and the percentage of fat mass was obtained by difference. This procedure was performed in all groups of cats at baseline and T60. 

### 5.5. Complete Blood Count and Biochemical Profile

Cats underwent 8 h of fasting and then 5 mL of blood were collected from the jugular or cephalic veins. For the CBC analysis, 0.5 mL was allocated in a tube containing EDTA. CBC was performed in an electronic counter (BC 5000 Vet, Shenzhen Mindray Animal Medical Technnology Co., Ltd, Shen Zhen, China). Red blood cell and platelet counts were determined by an electrical impedance method, the hemoglobin was determined by the colorimetric method and the differential leucocyte count was performed by laser flow cytometry. The other parameters were measured by calculation. Biochemical exams were performed in serum samples. For this, the exceeding blood was allocated in tubes with clot activator and centrifuged at 3000 rpm for 10 min. Serum creatinine, BUN, total protein, albumin, total calcium, total phosphorus, triglycerides, cholesterol, ALT, GGT, and ALP were analyzed by colorimetry methods. Serum concentrations of sodium, potassium, and chloride were determined by ion-selective electrode methodology. All biochemistry analyses were performed using a commercial kit in a biochemical analyzer (FUJI-DRI-CHEM NX 500i, Fujifilm^®^, São Paulo, Brazil) at PremieRpet^®^ Laboratory (Dourado, São Paulo, Brazil).

Serum SDMA was performed using the Catalyst SDMA IDEXX test (IDEXX^®^ Catalyst One Analyzer) at the Pet Care veterinary clinic (São Carlos, São Paulo, Brazil). Serum blood samples were sent to a reference laboratory (IDEXX laboratories, Inc., Westbrook, ME, USA) for PTH and iCa analysis. The PTH was measured at the Diagnostic Center for Population and Animal Health of Michigan State University (East Lansing, MI, USA) and iCa at the IDEXX Laboratories Brazil (São Paulo, São Paulo, Brazil).

### 5.6. Statistical Analysis

All variables were evaluated considering a general mixed linear model that included fixed effects for group (control group and cats IRIS stage 1 and 2), moments (T0, T30 and T60, or T0 and T60), interaction groups x moments, in addition to the random effects of animal within the group, age class, breed, and residue. The animals from each group were included in the analyses to accommodate the structure of repeated measurements in the same experimental units throughout the different evaluated moments. The covariance structures between repeated measures were evaluated by the Akaike’s information criterion (AIC) [28]. The assumptions of the analysis of variance models (normality and homogeneity of residuals) were performed simultaneously by conditional Studentized residuals analyses. The Tukey test was used as a procedure for comparing the means, aiming to maintain the fixed level of confidence, when significant results for the fixed effects of groups, moments or interaction groups x moments were verified. Statistical analyses were performed using the Statistical Analysis System, v. 9.4 (SAS^®^). Significance was set up at *p* < 0.05.

## Figures and Tables

**Table 1:** Nutrient and energy intake (mean ± standard error) of healthy cats (*n* = 10) and cats with CKD stage 1 (*n* = 6) and 2 (*n* = 9) included in the study according to the moments of evaluation and groups.

Treatment	Time Point			Mean		*p*-Value	
	T0	T30	T60		Treatment	Time Point	Treatment × Time
**Energy (kcal/BW^0.67^)**							
Control group	67.18 ± 6.23	73.26 ± 6.24	74.41 ± 6.24	71.62 ± 6.24	0.4487	0.0069	0.4632
IRIS stage 1	61.11 ± 8.00	64.50 ± 8.00	67.98 ± 8.00	64.53 ± 8.00			
IRIS stage 2	63.90 ± 6.16	63.87 ± 6.16	67.04 ± 6.16	64.94 ± 6.16			
Mean	64.06 ± 5.76 ^b^	67.21 ± 5.76 ^a^	69.81 ± 5.76 ^a^	-			
**Crude protein (g/BW^0.67^)**							
Control group	6.70 ± 0.56	6.02 ± 0.56	6.23 ± 0.56	6.32 ± 0.55	0.4467	<0.0001	0.8145
IRIS stage 1	5.98 ± 0.71	5.38 ± 0.71	5.68 ± 0.71	5.68 ± 0.69			
IRIS stage 2	6.25 ± 0.56	5.33 ± 0.56	5.60 ± 0.56	5.73 ± 0.55			
Mean	6.31 ± 0.56 ^a^	5.58 ± 0.56 ^b^	5.84 ± 0.56 ^b^	-			
**Phosphorus (g/BW^0.67^)**							
Control group	0.19 ± 0.01 ^a,A^	0.08 ± 0.01 ^b,A^	0.08 ± 0.01 ^b,A^	0.12 ± 0.01	0.4330	<0.0001	0.0009
IRIS stage 1	0.16 ± 0.01 ^a,B^	0.07 ± 0.01 ^b,A^	0.08 ± 0.01 ^b,A^	0.10 ± 0.01			
IRIS stage 2	0.17 ± 0.01 ^a,B^	0.07 ± 0.01 ^b,A^	0.08 ± 0.01 ^b,A^	0.11 ± 0.01			
Mean	0.17 ± 0.01	0.08 ± 0.01	0.08 ± 0.01	-			

BW, body weight; Control group, healthy cats. ^a,b^: Means on the same row and followed by the same lowercase letter did not differ (*p* > 0.05). ^A,B^: Means on the same column and followed by the same uppercase letter did not differ (*p* > 0.05).

**Table 2:** Body weight and body condition score (mean ± standard error) of healthy cats (*n* = 10) and cats with CKD stages 1 (*n* = 6) and 2 (*n* = 9) included in the study according to the moments of evaluation and groups.

Treatment	Time Point			Mean		*p*-Value	
	T0	T30	T60		Treatment	Time Point	Treatment × Time
**Body weight (kg)**							
Control group	4.92 ± 0.53 ^a,A^	4.89 ± 0.53 ^a,A^	4.90 ± 0.53 ^a,A,B^	4.90 ± 0.53	0.1927	0.0220	0.0106
IRIS stage 1	5.44 ± 0.63 ^a,A^	5.39 ± 0.63 ^a,A^	5.43 ± 0.63 ^a,A^	5.42 ± 0.63			
IRIS stage 2	4.76 ± 0.57 ^a,A^	4.63 ± 0.57 ^b,A^	4.47 ± 0.57 ^c,B^	4.62 ± 0.57			
Mean	5.04 ± 0.50	4.97 ± 0.50	4.94 ± 0.50	-			
**Body condition score**							
Control group	5.59 ± 0.37 ^a,A^	5.57 ± 0.37 ^a,A^	5.47 ± 0.37 ^a,A^	5.54 ± 0.37	0.4964	0.0012	0.0016
IRIS stage 1	6.15 ± 0.47 ^a,A^	6.14 ± 0.47 ^a,A^	6.15 ± 0.47 ^a,A^	6.14 ± 0.47			
IRIS stage 2	5.75 ± 0.42 ^a,A^	5.64 ± 0.42 ^a,A^	5.09 ± 0.42 ^b,A^	5.50 ± 0.42			
Mean	5.83 ± 0.26	5.79 ± 0.26	5.57 ± 0.26	-			

Control group, healthy cats. ^a,b,c^: Means on the same row and followed by the same lowercase letter did not differ (*p* > 0.05). ^A,B^: Means on the same column and followed by the same uppercase letter did not differ (*p* > 0.05).

**Table 3:** Muscle mass score and body composition (mean ± standard error) of healthy cats (*n* = 10) and cats with CKD stages 1 (*n* = 6) and 2 (*n* = 9) included in the study according to the moments of evaluation and groups.

Treatment	Time Point			Mean		*p*-Value	
	T0	T30	T60		Treatment	Time Point	Treatment × Time
**Muscle mass score**							
Control group	2.83 ± 0.14	2.84 ± 0.14	2.84 ± 0.14	2.84 ± 0.14 ^A^	0.0008	0.2131	0.1341
IRIS stage 1	3.00 ± 0.18	3.00 ± 0.18	3.00 ± 0.18	3.00 ± 0.18 ^A^			
IRIS stage 2	2.38 ± 0.15	2.38 ± 0.15	2.16 ± 0.15	2.31 ± 0.15 ^B^			
Mean	2.75 ± 0.12	2.75 ± 0.12	2.6 ± 0.12	-			
**Lean mass(kg)**							
Control group	3.98 ± 0.41	NM	3.91 ± 0.41	3.95 ± 0.40	0.1352	0.4559	0.2823
IRIS stage 1	3.94 ± 0.46	NM	4.04 ± 0.46	3.99 ± 0.46			
IRIS stage 2	3.50 ± 0.41	NM	3.26 ± 0.41	3.38 ± 0.41			
Mean	3.81 ± 0.36	NM	3.74 ± 0.36	-			
**Fat mass (kg)**							
Control group	1.42 ± 0.26	NM	1.13 ± 0.26	1.27 ± 0.24	0.1409	0.1887	0.5326
IRIS stage 1	1.90 ± 0.31	NM	1.81 ± 0.31	1.86 ± 0.30			
IRIS stage 2	1.30 ± 0.25	NM	1.28 ± 0.25	1.29 ± 0.24			
Mean	1.54 ± 0.20	NM	1.41 ± 0.20	-			

Control group, healthy cats; NM, not measured. ^A,B:^ Means on the same column and followed by the same uppercase letter did not differ (*p* > 0.05).

**Table 4:** Mean serum concentration (± standard error of mean) of total protein, creatinine, blood urea nitrogen, total calcium, ionized calcium, phosphorus, sodium, chloride, serum alkaline phosphatase, alanine aminotransferase, cholesterol, parathyroid hormone, and symmetric dimethylarginine of healthy cats (*n* = 10) and cats with CKD stages 1 (*n* = 6) and 2 (*n* = 9) included in the study according to the groups.

Variables	Reference Range	Control Group (*n* = 10)	IRIS Stage 1 (*n* = 6)	IRIS Stage 2 (*n* = 9)		*p*-Value	
					Treatment	Time Point	Treatment × Time
Total protein (g/dL)	5.7–7.8	7.92 ± 0.22	8.03 ± 0.25	7.95 ± 0.23	0.9185	0.0026	0.9368
Creatinine (mg/dL)	<1.6	1.30 ± 0.13 ^b^	1.21 ± 0.16 ^b^	1.87 ± 0.14 ^a^	0.0012	0.1284	0.1848
BUN (mg/dL)	17.6–32.8	24.00 ± 2.18 ^b^	22.67 ± 2.48 ^b^	34.18 ± 2.31 ^a^	0.0002	0.0811	0.4588
Ca (mg/dL)	8.8–11.9	9.69 ± 0.12 ^b^	10.25 ± 0.17 ^a^	10.18 ± 0.14 ^a^	0.0149	0.0024	0.3613
iCa (mmol/L)	1.0–1.4	1.27 ± 0.03	1.30 ± 0.02	1.30 ± 0.02	0.7569	0.3136	0.6607
Phosphorus (mg/dL)	2.6–6.0	5.76 ± 0.22 ^a^	4.88 ± 0.26 ^b^	5.50 ± 0.23 ^a,b^	0.0210	0.1253	0.5378
Sodium (mEq/L)	147–156	153.82± 0.68	154.24 ± 0.90	154.64 ± 0.75	0.5862	<0.0001	0.3073
Chlorine (mEq/L)	107–120	117.82 ± 0.98	117.87 ± 1.25	119.28 ± 1.08	0.2305	<0.0001	0.5265
ALP (U/L)	9–53	26.99 ± 5.11	32.94 ± 6.28	36.58 ± 5.69	0.4060	0.0099	0.2783
ALT (U/L)	22–84	63.03 ± 10.25	56.68 ± 13.41	77.38 ± 11.79	0.4888	0.0096	0.5270
Cholesterol (mg/dL)	89–176	146.39 ± 17.24	205.58 ± 20.18	176.90 ± 18.63	0.0541	<0.0001	0.5688
PTH (Mmol/L)	0.4–2.5	0.78 ± 0.16	0.79 ± 0.15	0.58 ± 0.15	0.4864	0.0151	0.0830
SDMA (µg/dL)	<18	10.16 ± 1.49	7.91 ± 1.96	12.26 ± 1.61	0.0594	0.0103	0.3137

BUN, blood urea nitrogen; Ca, total calcium; iCa, ionized calcium; ALP, alkaline phosphatase; ALT, alanine aminotransferase; PTH, parathyroid hormone; SDMA, symmetric dimethylarginine. ^a,b:^ Means on the same row and followed by the same lowercase letter did not differ (*p* > 0.05).

**Table 5:** Diets composition (per 100 kcal) and ingredients ^1^ according to the manufacturer.

Nutrients (g/100 kcal)	Senior Diet ^2^	Renal Test Diet ^3^
Protein	10.34	8.68
Fat	5.23	3.94
Crude fiber	0.62	0.41
Ash	1.87	1.08
Calcium	0.32	0.13
Phosphorus	0.29	0.12
Ca/P ratio	1.12	1.08
Potassium	0.17	0.22
Sodium	0.20	0.08
Omega-3	0.10	0.28
Metabolizable energy (kcal/kg)	3.920	4.353 *
**Essential amino acids**		
Arginine	0.70	0.48
Phenylalanine	0.49	0.36
Histidine	0.26	0.18
Isoleucine	0.42	0.37
Leucine	0.94	0.79
Lysine	0.59	0.49
Methionine	0.28	0.17
Taurine	0.07	0.05
Threonine	0.41	0.35
Tryptophan	0.08	0.09
Valine	0.52	0.46

^1^ Ingredients: ^2^ PremieR Gatos Castrados Acima dos 12 anos: poultry meal, pork protein isolate, corn gluten meal, egg product, broken rice, beet pulp, oat groats, chicken fat, soy oil, fish oil, hydrolyzed poultry and pork, sodium chloride, potassium chloride, antioxidants butylated hydroxyanisole and butylated hydroxytoluene, betaine, L-carnitine, L-lysine, fructooligosaccharide, mannooligosaccharide, sugarcane fiber, acidifying additive, chondroitin sulfate, glucosamine sulfate, taurine, Yucca schidigera extract, dried brewer’s yeast, vitamin and mineral premix; ^3^ Renal test diet: albumin, hydrolyzed chicken meal, poultry meal, pork protein isolate, corn gluten meal, egg product, soy protein isolate, barley, cassava flour, ground whole corn, soy lecithin, broken rice, beet pulp, poultry fat, pork fat, fish oil, calcium carbonate, potassium chloride, potassium citrate, fructooligosaccharide, galactooligosaccharide, mannooligosaccharide, sugarcane fiber, acidifying additive, antioxidants butylated hydroxyanisole and butylated hydroxytoluene, DL-methionine, L-lysine, magnesium oxide, calcium sulfate, vitamin and mineral premix; * Metabolizable energy of the diet previously calculated in a metabolism assay at the PremieRpet^®^ Nutritional Development Center.

## Data Availability

The data presented in this study are available on request from the corresponding author.

## Abbreviations

ALP: alkaline phosphatase; ALT: alanine aminotransferase; BC: body composition; BCAA: branched-chain amino acids; BCS: body condition score; BUN: blood urea nitrogen; BW: body weight; Ca: total calcium; iCa: ionized calcium; CBC: complete blood count; CKD: chronic kidney disease; GGT: gamma glutamyl transferase; LBM: lean body mass; ME: metabolizable energy; MER: maintenance energy requirements; MM: muscle mass; MMS: muscle mass score; PTH: parathyroid hormone; PUFAs: polyunsaturated fatty acids; SDMA: symmetric dimethylarginine; SBP: systolic blood pressure; sCr: serum creatinine.

## References

[1] Polzin D.J., Ettinger S.J., Feldman E.C., Côté E. (2017). Chronic Kidney Disease. Textbook of Veterinary Internal Medicine: Diseases of the Dog and the Cat.

[2] Forrester S.D., Adams L.G., Allen T.A., Hand M.S., Thatcher C.D., Remillard R.L., Roudebush P., Novotny B.J. (2010). Chronic Kidney Disease. Small Animal Clinical Nutrition.

[3] Polzin D.J., Bartges J., Polzin D.J. (2011). Chronic Kidney Disease. Nephrology and Urology of Small Animals.

[4] Ross S.J., Bartges J., Polzin D.J. (2011). Azotemia and Uremia. Nephrology and Urology of Small Animals.

[5] Segev G., Gram W.D., Milner R.J., Lobetti R. (2018). Chronic Kidney Disease. Chronic Disease Management for Small Animals.

[6] Jacob F., Polzin D.J., Osborne C.A., Allen T.A., Kirk C.A., Neaton J.D., Lekcharoensuk C., Swanson L.L. (2002). Clinical Evaluation of Dietary Modification for Treatment of Spontaneous Chronic Renal Failure in Dogs. J. Am. Vet. Med. Assoc..

[7] Plantinga E.A., Everts H., Kastelein A.M.C., Beynen A.C. (2005). Retrospective Study of the Survival of Cats with Acquired Chronic Renal Insufficiency Offered Different Commercial Diets. Vet. Rec..

[8] Elliott J., Rawlings J.M., Markwell P.J., Barber P.J. (2000). Survival of Cats with Naturally Occurring Chronic Renal Failure: Effect of Dietary Management. J. Small Anim. Pract..

[9] Scherk M.A., Laflamme D.P. (2016). Controversies in Veterinary Nephrology: Renal Diets Are Indicated for Cats with International Renal Interest Society Chronic Kidney Disease Stages 2 to 4: The Con View. Vet. Clin. N. Am. Small Anim. Pract..

[10] Chacar F.C., Kogika M.M., Zafalon R.V.A., Brunetto M.A. (2020). Vitamin d Metabolism and Its Role in Mineral and Bone Disorders in Chronic Kidney Disease in Humans, Dogs and Cats. Metabolites.

[11] Polzin D.J., Osborne C.A., Adams L.G. (1991). Effect of Modified Protein Diets in Dogs and Cats with Chronic Renal Failure: Current Status. J. Nutr..

[12] Polzin D.J., Osborne C.A., Ross S., Jacob F. (2000). Where Are We Now? In What Direction Are We Headed?. Clin. Sci..

[13] IRIS (2019). Staging of CKD.

[14] Cupp C.J., Kerr W. (2010). Effect of Diet and Body Composition on Life Span in Aging Cats. Companion Animal Nutrition Summit: Focus on Gerontology.

[15] Freeman L.M., Lachaud M.P., Matthews S., Rhodes L., Zollers B. (2016). Evaluation of Weight Loss over Time in Cats with Chronic Kidney Disease. J. Vet. Intern. Med..

[16] Hall J.A., Fritsch D.A., Jewell D.E., Burris P.A., Gross K.L. (2019). Cats with IRIS Stage 1 and 2 Chronic Kidney Disease Maintain Body Weight and Lean Muscle Mass When Fed Food Having Increased Caloric Density, and Enhanced Concentrations of Carnitine and Essential Amino Acids. Vet. Rec..

[17] Boyd L.M., Langston C., Thompson K., Zivin K., Imanishi M. (2008). Survival in Cats with Naturally Occurring Chronic Kidney Disease (2000–2002). J. Vet. Intern. Med..

[18] Chakrabarti S., Syme H.M., Elliott J. (2012). Clinicopathological Variables Predicting Progression of Azotemia in Cats with Chronic Kidney Disease. J. Vet. Intern. Med..

[19] Laflamme D.P. (1997). Development and Validation of a Body Condition Score System for Cats: A Clinical Tool. Feline Pract..

[20] FEDIAF (2021). Nutritional Guidelines for Complete and Complementary Pet Food for Cats and Dogs.

[21] Tom A., Nair K.S. (2006). Assessment of Branched-Chain Amino Acid Status and Potential for Biomarkers. J. Nutr..

[22] Ephraim E., Jewell D.E. (2021). High Protein Consumption with Controlled Phosphorus Level Increases Plasma Concentrations of Uremic Toxins in Cats with Early Chronic Kidney Disease. J. Food Sci. Nutr..

[23] NRC (2006). Nutrient Requirement of Dogs and Cats.

[24] King J.N., Tasker S., Gunn-Moore D.A., Strehlau G. (2007). Prognostic factors in cats with chronic kidney disease. J. Vet. Intern. Med..

[25] Polzin D.J. (2011). Chronic Kidney Disease in Small Animals. Vet. Clin. N. Am. Small Anim. Pract..

[26] Michel K.E., Anderson W., Cupp C., Laflamme D.P. (2011). Correlation of a feline muscle mass score with body composition determined by dual-energy X-ray absorptiometry. Br. J. Nutr..

[27] Barber P.J., Elliott J. (1998). Feline Chronic Renal Failure: Calcium Homeostasis in 80 Cases Diagnosed between 1992 and 1995. J. Small Anim. Pract..

[28] Akaike H. (1973). Information Theory and an Extension of the Maximum Likelihood Principle. Proceedings of the 2nd International Symposium on Information Theory.

